# A Temporal Network Approach to Paranoia: A Pilot Study

**DOI:** 10.3389/fpsyg.2020.544565

**Published:** 2020-09-18

**Authors:** Alba Contreras, Carmen Valiente, Alexandre Heeren, Richard Bentall

**Affiliations:** ^1^Department of Personality, Assessment and Clinical Psychology, Complutense University of Madrid, Madrid, Spain; ^2^Psychological Sciences Research Institute, Université Catholique de Louvain, Louvain-la-Neuve, Belgium; ^3^Institute of Neuroscience, Université Catholique de Louvain, Brussels, Belgium; ^4^Department of Psychology, University of Sheffield, Sheffield, United Kingdom

**Keywords:** paranoia, sadness, self-esteem, experience sampling methodology, temporal network analysis, intensive time-series

## Abstract

Paranoid beliefs have been conceptualized as a central psychological process linked to schizophrenia and many mental disorders. Research on paranoia has indicated that it is pivotal to consider not only levels but also dynamic aspects of incriminated related mechanisms over time. In the present study, we conceptualized paranoia as a system of interacting elements. To do so, we used temporal network analysis to unfold the temporal dynamics between core psychological paranoia-related mechanisms, such as self-esteem, sadness, feeling close to others, and experiential avoidance. Time-series data of 23 participants with high scores in paranoia and/or interpersonal sensitivity were collected *via* experience sampling methodology (ESM). We applied a multilevel vector autoregressive (mlVAR) model approach and computed three distinct and complementary network models (i.e., contemporaneous, temporal, and between-subject) to disentangle associations between paranoia-related mechanisms in three different time frames. The contemporaneous model indicated that paranoia and sadness co-occurred within the same time frame, while sadness was associated with both low self-esteem and lack of closeness to others. The temporal model highlighted the importance of feeling close to others in predicting low paranoia levels in the next time frame. Finally, the between-subject model largely replicated an association found in both contemporaneous and temporal models. The current study reveals that the network approach offers a viable data-driven methodology for elucidating how paranoia-related mechanisms fluctuate over time and may determine its severity. Moreover, this novel perspective may open up new directions toward identifying potential targets for prevention and treatment of paranoia-related problems.

## Introduction

Paranoia has been defined as “unfounded thoughts that others are deliberately intending to cause harm” (Murphy et al., 2018). Though traditionally linked to schizophrenia and other psychotic disorders (Jorgensen and Jensen, 1994; Bentall et al., 2001), paranoid beliefs are common in the general population (Freeman, 2006) and exist on a continuum (Elahi et al., 2017). At the clinical level, paranoia has been conceptualized as a transdiagnostic feature associated with affective dysregulations and emotional disorders (Bentall et al., 2009). In this way, paranoia constitutes a viable target for clinical interventions, not only among people with psychotic disorders but also across a wide range of mental disorders (Freeman, 2007; Lincoln et al., 2013).

Yet, uncertainties still abound regarding the underlying mechanisms driving paranoid beliefs (Bentall et al., 2001; Freeman et al., 2002). Prominent models of paranoia highlight self-esteem as a core process of paranoia (Bentall et al., 2001; Freeman et al., 2002). According to the self-serving bias theory, paranoia fulfills a defensive function blaming others of negative events (Bentall et al., 2001). Conversely, paranoid beliefs have also been viewed as a direct reflection of individual’s negative self-schemas (Freeman et al., 2002). Without alluding to a defensive process as an explanation, this perspective suggest that individuals’ social and emotional difficulties would simply correlate with low self-esteem and depression (Freeman, 2007). Nonetheless, results regarding the role of self-esteem in paranoia are inconsistent. Supporting Freeman’s proposal, some authors have evidenced that people with paranoid beliefs exhibited lower self-esteem than those without such beliefs (Kesting and Lincoln, 2013). On the other hand, although some predictions of the defense model are not supported (i.e., explicit self-esteem preserved), a recent meta-analysis has revealed that, based on studies relying on experience sampling methodology (ESM, an intensive longitudinal research methodology that involves asking participants to report on their thoughts, feelings, behaviors, and/or environment on multiple occasions over time), instability of self-esteem is clearly associated with increased severity of paranoia (Murphy et al., 2018). Uncertainty thus remains regarding the precise role of self-esteem in paranoia.

In addition to self-esteem, other processes have also been associated with the onset and maintenance of paranoid beliefs. First, negative affect is common in people with paranoid thinking (Freeman et al., 2011; Hartley et al., 2013; Vorontsova et al., 2013). In fact, the presence of depressive symptoms, in particular, has been associated with a 7-fold-increased risk of experiencing the most severe form of paranoid beliefs (Freeman et al., 2011; Freeman and Garety, 2014). Previous ESM research has also revealed that depression was associated with the duration of the paranoid episodes (Thewissen et al., 2011). Second, people with paranoia exhibit higher levels of dysfunctional emotional regulation strategies such as experiential avoidance (EA), that is, the unwillingness to remain in contact with particular private negative experiences (e.g., bodily sensations, thoughts, and emotions) and attempts to eliminate them (Hayes et al., 2004; Udachina et al., 2009). Interestingly, the relation between paranoia and EA has been found to be partially mediated by low self-esteem in time-series studies (Udachina et al., 2014). In favor of the self-serving bias theory (Bentall et al., 2001), people with paranoia exhibited greater life satisfaction when they have greater experiential avoidance and low insight, suggesting EA might serve as a “defensive strategy” (Valiente et al., 2015). Finally, a third critical paranoia-related process is cognitive schemas about others (Fowler et al., 2006). Research has shed light about the association between paranoia with a negative view of others (Lamster et al., 2017) as well as with subjective perception of social exclusion (Westermann et al., 2012). One way of construing these observations may be within the attachment framework (Berry et al., 2019), although it is not exempt of critics as it is often assessed in adulthood, whereas the original theory was based on studies of children and their caregivers (Bartholomew and Shaver, 1998; Gumley et al., 2014). Notwithstanding this limitation, a specific relationship between insecure attachment and paranoia has been observed in multiple clinical, population, and analogue studies, which might, in turn, be reflected in interpersonal problems (Gumley et al., 2014; Berry et al., 2019; Lavin et al., 2019).

Recently, the network approach has emerged in psychopathology (Borsboom and Cramer, 2013; for a review, see Contreras et al., 2019; Robinaugh et al., 2019). From this perspective, mental disorders are conceptualized as complex network systems, wherein elements (i.e., symptoms or psychological processes) interact and evolve over time (Borsboom, 2017). Accordingly, one may expect that the aforementioned paranoia-related processes (i.e., self-esteem, sadness, experiential avoidance, and closeness to others) are embedded within a network system, wherein they trigger one another over time.

Previous network research has relied on cross-sectional models to study psychosis (Isvoranu et al., 2017, 2018; van Rooijen et al., 2017). However, only a few studies included paranoia as an element in the network (Isvoranu et al., 2016; Bell and O’Driscoll, 2018; Hajdúk et al., 2019). Yet, their findings were heterogeneous (for a discussion, see Contreras et al., 2019). Moreover, although this nascent field is promising, cross-sectional designs preclude strong inference regarding the causal (e.g., Maurage et al., 2013) as well as the temporal relationships among paranoia-related processes (e.g., Bos et al., 2017). Indeed, whereas these network models are good at describing associations between the average scores on the variables of interest, they fall short of explaining how these variables dynamically trigger each other over time. Hereafter, we believe conceptualizing paranoia-related processes as a temporal network, rather than restricting it to cross-sectional associations between processes, may offers clues to generating new hypotheses about the temporal dynamic interplay of the paranoia-related processes.

A dynamic conceptualization of paranoia-related processes can be done by generating network models from intensive time-series data collected *via* ESM (for a review, see Myin-Germeys et al., 2018). Some ESM studies have shed light on time-lagged associations between paranoia and core processes like self-esteem, negative affect, or experiential avoidance (Thewissen et al., 2008, 2011; Udachina et al., 2009, 2014; Ben-Zeev et al., 2011; Kramer et al., 2014). Although the aforementioned variables have been well established in previous ESM research, a network perspective provides a different approach. Temporal network analysis call upon a multilevel vector autoregressive (mlVAR) approach that allows the estimations of three types of networks taking into account three different time frames (i.e., the same measurement time, different measurement occasion, and 1-week average; Epskamp et al., 2018d). The advantage of multilevel temporal network model is that it offers the possibility of considering the intra‐ and the inter-individual level of information and creates network models that control for all other variables and temporal effects (e.g., Epskamp et al., 2018a,d). In this way, by using an mlVAR network approach in combination to the ESM data, one may disentangle the temporal sequence of the dynamic interaction between more than one variable of interest (Bringmann et al., 2016; Epskamp et al., 2018a,d; Hoorelbeke et al., 2019). Although there is a growing interest in applying temporal network analysis in psychology, only one single case study has, to date, included paranoia as an element (Bak et al., 2016).

The aim of the current study was thus to examine the temporal dynamics of the aforementioned theory-driven paranoia-related processes (i.e., self-esteem, feeling of closeness to the others, experiential avoidance, and sadness) by conducting temporal network analyses on ESM data.

## Materials and Methods

### Open Science Practice

The de-identified data, ESM’s items, and R code are publicly available *via* the Open Science Framework (OSF) and can be accessed at https://osf.io/7tk4b/.

### Participants

Participants were recruited from a larger randomized controlled trial registered at *ClinicalTrials.gov* ^1^, aiming at testing the impact of psychological group intervention for people with paranoid tendencies (Valiente et al., 2019). They were attending the Psychology Clinic of the Complutense University of Madrid for clinical psychological distress (i.e., mood, anxiety, interpersonal, or nonspecified problems), and they were referred by their therapist. The current paper reports findings from the assessment phase that preceded the treatment protocol.

Eligibility criteria were as follows: (a) be over 18 years old and (b) scoring at least one SD above the population mean in the subscales for paranoid ideation and/or interpersonal sensitivity of the validated Spanish version of the Symptom Checklist 90-Revised (SCL-90-R; Derogatis, 2001). This is a widely used scale, especially as a screening tool, to assess psychological and psychopathological symptoms in both clinical and normal populations (Derogatis, 2001). The latter criterion was used to broaden the range of paranoid experience included in the study, as previous research has revealed that interpersonal sensitivity is associated with paranoid thinking (Bebbington et al., 2013; Isvoranu et al., 2016; Meisel et al., 2018), and paranoid ideation can be considered as an extension of such concerns (Freeman and Garety, 2014). Following the screening procedure, 64 patients were enrolled in the study and thus participated in the ESM assessment. Of this sample, we only included data from participants providing over 21 valid responses (i.e., 1/3 of potential total number of responses); a cutoff based on prior research combining network analysis and ESM methodology (Aalbers et al., 2019; Greene et al., 2019). The resulting 23 participants who were included in the analyses (82.6% females) completed an average of 28.48 measurements (*SD* = 6.58). Note that included and excluded participants did not differ on any demographic or clinical variables (see Table S1 in the Supplementary Materials available on the OSF at https://osf.io/378q4/). Demographic and clinical characteristics are depicted in Table 1.

**Table 1:** Sample characteristics.

	Participants *n* = 23
*Demographic characteristics*	
Age in years, mean (SD)	23.78 (6.17)
Sex: women, *n* (%)	19 (82.6)
Single status, *n* (%)	22 (95.7)
Education, *n* (%)	
Secondary school	3 (13)
Post-secondary	20 (87)
Employed, *n* (%)	
Unemployed	15 (65.2)
Part-time employment	5 (21.7)
Full-time employment	3 (13)
*Clinical characteristics*	
SCL-90-R paranoid ideation, mean (SD)	1.24 (0.92)
SCL-90-R interpersonal susceptibility, mean (SD)	1.57 (0.86)
SCL-90-R anxiety, mean (SD)	1.10 (0.46)
SCL-90-R depression, mean (SD)	1.93 (0.74)
*Participants diagnosis: n* (%)	
Not meeting criteria for diagnosis	9 (39.1)
Major depression disorder	5 (21.7)
Anxiety disorder (includes PD and GAD)	3 (13)
Posttraumatic stress disorder	2 (8.7)
Dysthymia	3 (13)
Trichotillomania	1 (1.3)
*ESM observations*	
Number of completed observations, mean (SD)	28.48 (6.58)
Number of missing beeps, mean (SD)	30.83 (13.90)
Number of missing data, mean (SD)	10.70 (12.95)

SCL-90-R, symptom checklist 90-R (Cronbach’s *α* = 0.79–0.90); SD, standard deviation; PD, panic disorder; GAD, generalized anxiety disorder; number of missing beeps = the final amount of missing notifications due to technical problems; number of missing data = notifications that participants did not response.

### ESM Measures

We used a time-contingent ESM design (Myin-Germeys et al., 2018), whereby participants received 10 notifications a day between 9:00 AM and 10:00 PM over a 7-day period. We used a stratified schedule wherein, each day, 10 notifications were delivered between intervals of, at least, 30 min between each signal. Signals timed-out 15 min after being sent (Delespaul, 1995).

The study was part of a larger ESM protocol, which included 33 items, which took an average of 5 min to complete. For the present study, we only focused on paranoia-related processes, which include seven items: (a) *Negative affect*, we use one item to measure sadness (“At this moment, I feel sad”). This ESM item has been previously used to study negative affect (e.g., Palmier-Claus et al., 2011); (b) *Self-esteem*, we adapted two items from the Rosenberg Self-esteem Scale (RSES; Rosenberg, 1989; “At this moment, I feel useful” and “At this moment, I feel I can manage issues well”). The internal consistency of this scale was *α* = 0.745; (c) *Closeness to others*, as persecutory beliefs are also associated with negative beliefs about others as well as social exclusion, we designed one item to assess how close individuals perceived others (“At this moment, I feel close to others”); (d) *Experiential avoidance*, as an emotional regulation strategy, we adapted one item from the Acceptance and Action Questionnaire-II (AAQ-II; Hayes et al., 2004; “Since the last beep, I have tried to avoid negative thoughts and feelings”); and (e) *Paranoid beliefs*, following the Persecutory Ideation Questionnaire (PIQ; McKay et al., 2006), we adapted three items (“Since the last beep, I have had the impression that I cannot trust people,” “I have had the impression that people have tried to harm me,” and “I have had the impression that people have criticized me”). The internal consistency of this scale was *α* = 0.737; self-esteem and paranoid ideation were computed using the average of their corresponding items (see section “Identifying Redundant Items” in the Supplementary Materials). All items were presented on a 9-point Likert-type scale, and a careful translation was carried out, following the latest indications (e.g., Wild et al., 2005; for English and Spanish versions of the ESM items, see Table S2 in the Supplementary Materials available on the OSF at https://osf.io/378q4/).

### Procedure

As a part of a broader clinical trial project, the ESM data reported here was collected during the baseline assessment of the trial (Valiente et al., 2019). At the outset, we used Personal Analytics Companion application (PACO APP; https://pacoapp.com), a free and open-source application for building and running ESM studies. However, this application ceased to be accessible in midway through the research, at which point we switched to the Qualtrics platform^2^. Note that in both platforms everything was made identical and there were no significant differences in terms of demographic and clinical characteristics between people who received the ESM procedure *via* the first platform vs. the second platform (see Table S3 in the Supplementary Materials). An initial one-on-one instructional session was organized with each participant. During this session, an instructor (i.e., the first author of this paper) demonstrated the ESM-platform use and turned on the notification’s parameters of the participant’s mobile phone. Participants were given an email address to contact in case of questions or technical problems with the application. After the completion of the ESM, participants received information about their performance. In addition, after the completion of the RCT, the results of the study were available for the University Clinic staff as well as participants. The study was approved by the Complutense University of Madrid’s Institutional Review Board and conducted according to the Declaration of Helsinki. All participants provided written informed consent and were fully debriefed at the end of the study.

### Data Analysis

#### Assumptions Check

We used the Shapiro-Wilk normality test to test whether each variable was normally distributed. Multilevel vector autoregressive (VAR) analysis employed to analyze the data assumes that the mean and variance of a variable do not change as a function of time – i.e., the assumption of stationarity (Bringmann et al., 2016; Aalbers et al., 2019). To test this assumption, we used the Kwiatkowski-Phillips-Schmidt-Shin unit root test (KPSS Test for Stationarity) for each variable of each participant, as implemented in the R package *tseries* (Trapletti and Hornik, 2019).

#### Network Estimation and Visualization

To analyze the dynamic relationships between the variables, we used the VAR model on the ESM data, implemented in *mlVAR* R package (version 0.4.3; Epskamp et al., 2017). Within this model, we estimate three network structures (Epskamp et al., 2018d): (1) a contemporaneous network, which is a Gaussian graphical model (GGM) that depicts within-time-window edges (associations between nodes) corresponding to a multilevel partial correlation network, after controlling for temporal associations; (2) a temporal network, which is a directed network of regression coefficients that depicts the lagged associations between nodes from one measurement point to the next measurement point after controlling for all other variables at the previous measurement point; and (3) finally, a between-subject network, which is a GGM that depicts regularized partial correlations (after taking into account the remaining variables in the network) between individuals’ means during a specific period of time (Epskamp et al., 2018a,d). Thus, while the contemporaneous network model inform about association at the same time frame, the between-subject network model reveals, on average (i.e., 1-week assessment), the variance–covariance structure of participants’ means.

Decomposing the variance in these three distinct networks provides different but complementary insights in the covariation and potential dynamics of the constructs of interest. First, the contemporaneous network shows whether deviations from a person’s means on two variables predict one another at the same measurement occasion (Epskamp et al., 2018c; Greene et al., 2018). Second, the temporal network indicates whether a deviation from a person’s mean predicts a deviation from that person’s mean in another variable at the next measurement occasion (Bringmann et al., 2016; Epskamp et al., 2018c). Finally, the between-subject network mirrors the covariation between means of participants (Epskamp et al., 2018c; Greene et al., 2018) and, in this way, allows for comparison with previous cross-sectional studies (Epskamp et al., 2018c,d).

The mlVAR package calls upon the qgraph package (version 1.6.3; Epskamp et al., 2018b) to plot the estimated coefficients as graphical network models. For the contemporaneous and between-subject networks, we used the conservative “AND-rule” approach in retaining significant edges – that is, an edge was retained if both regressions, on which the edge was based, were significant (*α* = 0.05). For each network model, blue lines on the graphical representations show positive associations, whereas red lines show negative ones. The strength of the connectivity is represented by thickness of the edges. A thicker edge denotes a larger association. In the temporal network, arrows are used as edges to illustrate the direction of effects. Interpretations regarding centrality of nodes rely on visual inspection of the obtained network structures, given that standardized centrality indices are not ideal for multilevel VAR-models (Bringmann et al., 2016).

## Results

### Descriptive Statistics

Means and standard deviations of within-participant means and within-participant standard deviations of each variable are depicted in Table 2.

**Table 2:** Means and standard deviations of within-participant means and within-participant standard deviations for all ESM variables.

*ESM variable*	*M (SD)*	*SD (SD)*
Sad	3.92 (1.59)	1.73 (0.50)
Closeness to others (others)	4.73 (1.36)	1.59 (0.62)
Self-esteem (SE)	4.95 (1.12)	1.27 (0.40)
Experiential avoidance (EA)	4.21 (1.79)	1.77 (0.61)
Paranoia	2.01 (1.03)	0.78 (0.50)

M, mean; SD, standard deviation. All variables were measured using a 9-point Likert scale (range 0–9).

### Assumption Checks

Shapiro-Wilk tests indicated that no variable was normally distributed (see Table S4 in the Supplementary Materials available on the OSF at https://osf.io/378q4/). As we aimed to account for temporal variability, we conducted the analysis as planned regardless this assumption. KPSS unit root tests suggested stationary data for all variables in all participants.

### Network Estimation and Visualization

#### Contemporaneous Network

**Figure 1:**
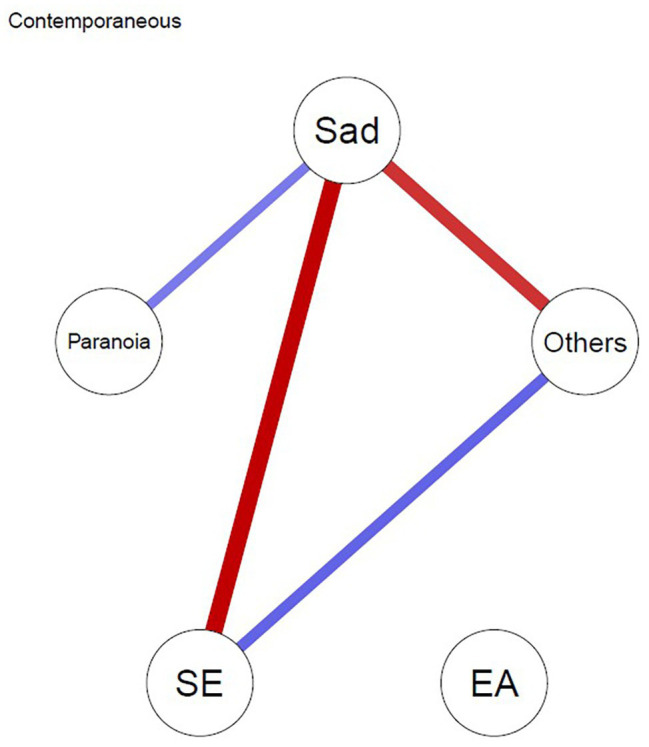
Contemporaneous network model. Edges represent associations between the variables within the same time frame after controlling for temporal associations. Blue lines depict positive associations and red lines depict negative associations between variables. SE, self-esteem; EA, experiential avoidance, Others = closeness to others.

Figure 1 depicts the contemporaneous network – i.e., the associations between the variables within the same time frame after controlling for all other temporal and contemporaneous relations. A few connections stand out. Paranoia and sadness are positively associated, suggesting that higher scores in paranoia are associated with higher of levels of sadness during the same time frame. Likewise, sadness is negatively associated with both self-esteem and feeling close to others. In other words, the higher the sadness, the lower the levels of self-esteem and feeling close to others at the same moment. Moreover, self-esteem and feeling of being close to others are positively associated. Finally, experiential avoidance is not connected to any other variable in this analysis.

#### Temporal Network

**Figure 2:**
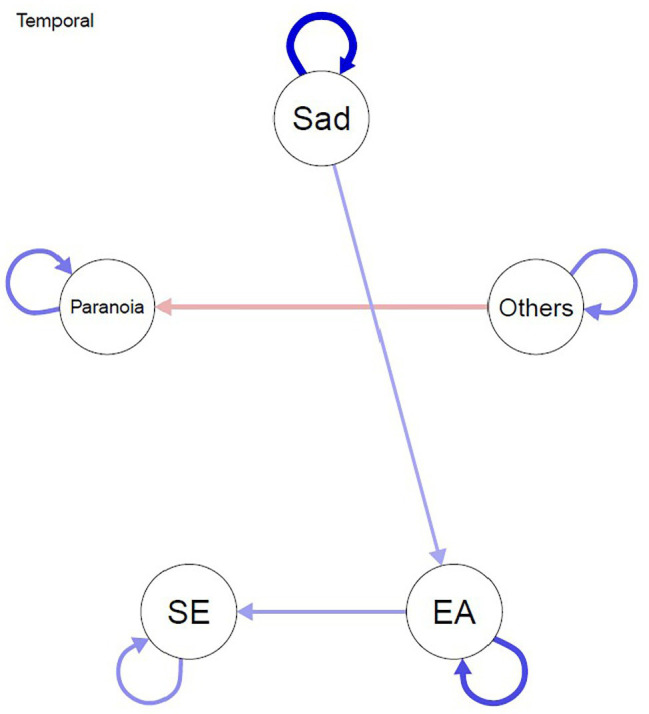
Temporal network model. Edges represent prediction between nodes from one measurement point to the next measurement point that remain after controlling for all other variables at the previous measurement point. Blue lines depict positive associations and red lines depict negative associations between ESM variables. SE, self-esteem; EA, experiential avoidance; Others = closeness to others.

Figure 2 depicts the temporal network, which represents the extent to which nodes predicted themselves (i.e., autoregression) and each other from one time frame (*t*) to the next time frame (*t* + 1). The arrow depicts the direction of the prediction, and this analysis is much more informative about potential causal mechanisms. Unsurprisingly, all nodes show positive autocorrelation over time; with sadness being particularly autoregressive, these findings simply show the relative stability of these variables over relatively short time frames. Much more importantly, feeling of being close to others negatively predicted paranoia at the next time point and sadness positively predicted experiential avoidance, which, in turn, positively predicted self-esteem at the next time point.

#### Between-Subject Network

**Figure 3:**
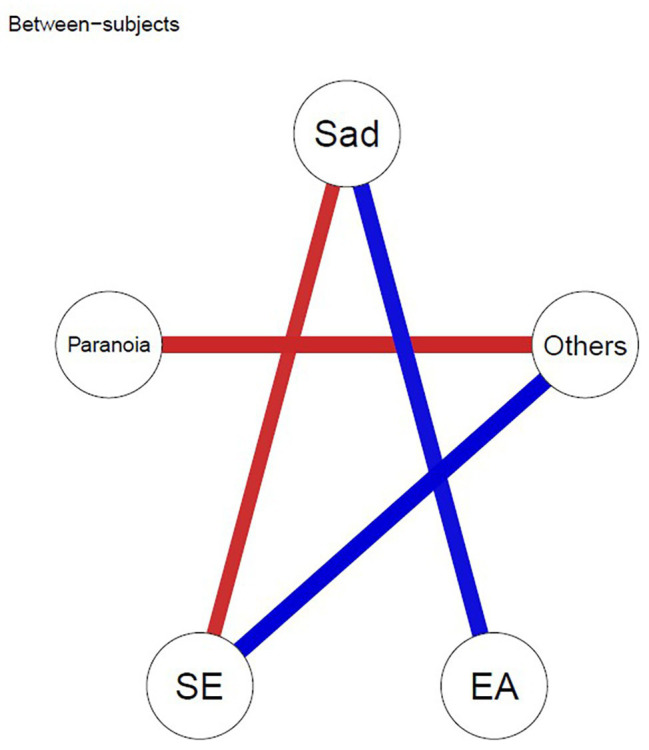
Between-subject network model. Edges represent correlations between intra-individual mean levels, after taking into account the remaining variables in the network. Blue lines depict positive associations, and red lines depict negative associations between ESM variables. SE, self-esteem; EA, experiential avoidance; Others = closeness to others.

The between-subject network shows the correlations between intra-individual mean levels of the nodes over the entire testing week. That is, the associations between individual’s means during the overall ESM week. As shown in Figure 3, the mean levels of closeness to others was negatively associated with the mean levels of paranoia and positively associated with mean levels of self-esteem. The mean level of sadness was negatively associated with the mean levels of self-esteem and positively associated with mean levels of experiential avoidance.

## Discussion

In this study, we aimed at unfolding the temporal interplay between sadness, experiential avoidance, self-esteem, feeling of being close to others, and paranoia during one ESM week among 23 participants with high scores in paranoia. We applied temporal network analysis and computed three network models to examine within‐ and between-individual differences over time. The contemporaneous and between-subject networks consider the way that the variables of interest covary, at the same time point and on average, respectively; the latter in effect replicates cross-sectional analyses previously reported in the literature but with longitudinal data (Epskamp et al., 2018c,d). The temporal network allows us to move closer to identifying the potential causal interplay between the variables by considering how events at one time point predicts what happens at the next. It is worth noting that, because of the timing of the ESM assessments, the temporal model can only detect potential causal associations that take place over a number of hours (Greene et al., 2018). Note also that all nodes show self-loops, indicating that all variables predict themselves at the next time frame, which may point to a degree of stability in the variables. Some previous ESM studies have used lagged data to identify how changes in specific variables lead to changes in others, for example how low self-esteem (Thewissen et al., 2008), experiential avoidance (Udachina et al., 2014), and momentary attachment insecurity (Sitko et al., 2016) relate to paranoia, but network analysis allows the interplay between all of these variables to be considered at the same time. Together, these networks provide insights into the dynamical nature of paranoid beliefs when taking into account different time frames.

Perhaps the most surprising finding is a negative one: paranoia was not directly related to self-esteem in any of the three networks. Hence, although there has been consistent evidence supporting the role of self-esteem in paranoia from previous studies (Kesting and Lincoln, 2013; Murphy et al., 2018), the current findings do not replicate this effect and therefore call into question psychological models which afford self-esteem a central role, for example the attributional model of paranoia proposed by Bentall et al. (2001). Several explanations could account for this unexpected lack of association, other than the possibility that it does not, in fact, exist.

First, the relationship between the two variables might have become nonsignificant when controlling for the remaining variables in the model. Sadness is a candidate variable in this respect. Both the contemporaneous and between-subject networks reported a negative association between sadness and self-esteem consistent with numerous previous time-series studies of both depression (Orth and Robins, 2013) and paranoia (Thewissen et al., 2011). Second, the association between paranoia and self-esteem might be mediated by other processes. In fact, our contemporaneous network model evidences that the relation between paranoia and self-esteem is conditionally independent, given the presence of sadness. These findings are consistent with previous cross-sectional research that shows associations between paranoia, low mood, and low self-esteem (e.g., Thewissen et al., 2011). This result indicates that sadness could have a mediating role on a small timescale, supporting previous findings that point to depression as a significant mediator in the relationship between paranoia and self-esteem (Ben-Zeev et al., 2009). Accordingly, the current findings can be interpreted as in line with the cognitive perspective of paranoia, which assumes that low self-esteem affects paranoia largely through depressed and anxious symptomatology (Freeman, 2016).

The most important positive finding of this study is that closeness to others is directly and negatively associated with paranoia in the between-subjects network and the findings from the temporal network show a similar effect, raising the possibility that this effect is causal. This observation aligns with an attachment framework for understanding paranoia (Berry et al., 2019) and previous studies showing that paranoia can be triggered by interpersonal factors such as negative interpersonal schemata (Lincoln et al., 2010), momentary attachment insecurity (Sitko et al., 2016), perceived social exclusion (Westermann et al., 2012), and impoverished social network (Gayer-Anderson and Morgane, 2013). Furthermore, our results reveal that closeness to others is positively associated with self-esteem in both the between-subject and the contemporaneous network. Again, these findings are completely consistent with previous cross-sectional research, which shows associations between positive beliefs about the self and secure attachment (Gayer-Anderson and Morgane, 2013).

Finally, the networks are informative about the role of experiential avoidance. Experiential avoidance refers to the need to avoid distressing mental contents, and has been hypothesized to play an important role in maintaining psychopathology (Hayes et al., 2004). Previous studies have reported high experiential avoidance in paranoid patients and non-patients. In studies with analogue (Udachina et al., 2009) and patient samples (Udachina et al., 2014), some positive direct effect of experiential avoidance on paranoia was found, as well as an indirect effect through lowered self-esteem; these findings were interpreted as paranoia arising from failed attempts to avoid negative thoughts about the self. In our analyses of the present data, however, no direct association between experiential avoidance and paranoia was found. Moreover, the association between experiential avoidance and sadness is consistent in the between-subjects and temporal network. In the temporal network, sadness provoked experiential avoidance, which in turn led to improved self-esteem, an effect that is entirely consistent with the original conceptualization of the experiential concept by Hayes et al. (2004). Overall, these findings suggest a complex relationship between experiential avoidance and mood but, at most, a distant and very indirect role for experiential avoidance in paranoid thinking.

### Clinical Implications

Our findings yield clinical implications. Overall, our results underscore the importance of taking into account the dynamic nature of psychological phenomena. Temporal network analysis might be useful to identify potential therapeutic target that may change the dynamic in the network. Specifically, based on the results from the temporal network model, it may be hypothesized that intervening on attachment-related cognitions may reduce paranoid thinking over time (Berry et al., 2019). It is possible that clinical effects will be enhanced by focusing on the positive aspect of social relations, instead of focusing on their deficit (Wykes et al., 2008). This idea is in line with the recent increased awareness in the need for a positive movement in psychology, focused on positive psychosocial factors and well-being (Jeste et al., 2017). Positive psychology intervention for psychosis have encouraged the enhancement of positive social relationships as a protective factor (Slade, 2010; Slade et al., 2016), and accordingly, our findings suggest that targeting interpersonal processes might be beneficial for people with vulnerability to paranoia.

### Limitations and Strengths

This is a pilot study, and several issues require further examination in follow-up research. First, the compliance to the ESM protocol was low, resulting in a small final sample size (*n* = 23). Although the number of measurements per person is satisfactory, the sample is modest for temporal network analysis and replications would benefit from a larger group of participants. Several explanations may explain this issue. To begin with, because the use of both ESM apps relies on internet access, one cannot exclude that whenever participants were not connected to a proper network, they were not properly notified and, in turn, missed the beeps (see Table S1 in the Supplementary Materials available on the OSF at https://osf.io/378q4/). Second, the length of the ESM protocol could have influenced the number of dropouts. Another potential explanation could be that people with high paranoid tendencies do not rely on devices or do not feel comfortable sharing their experiences *via* an app. However, although prior research has made efforts to cast light on potential predictors of compliance and did not found demographic or clinical variables to be related to lack of compliance (Hartley et al., 2014), the study of the adherence to an ESM-protocol among people with paranoid tendency has yet to be done.

Second, our three network models are low dimensional (i.e., few nodes relative to the number of participants). Network analyses, like any statistical tool, can only examine variables that are entered into a model. Therefore, though the current graphs are parsimonious with only five nodes and based on current prominent models of paranoia, there could be important variables left out. For instance, the external explanation for negative events and a distinction between explicit and implicit self-esteem are important components in one of the most influential model of paranoia (Bentall et al., 2001; Murphy et al., 2018). Thus, we encourage future research to compare empirical data model to theory models (Haslbeck et al., 2019; Heeren et al., 2020).

Third, as this sample was made of participants with paranoia vulnerability (i.e., subclinical population), the mean levels of paranoia are low. We consider that replications of the current study in a population with higher paranoid severity are needed. Fourth, our data did not meet normality assumptions. Such an approach is common in psychological sciences and has been reported in previous temporal network studies (e.g., Aalbers et al., 2019). Some authors have highlighted that assuming normally distributed parameters can be problematic because it imposes that subjects cannot differ on the structure of the network (Epskamp et al., 2018d). However, it is still unclear how robust time-series analysis is to these violations, and results should be interpreted cautiously. We consider this issue to be an essential direction for future work on temporal network analysis. In addition, it is pivotal to state that the obtained results are useful for generating hypotheses about the causality of paranoia-related processes, but not sufficient to draw robust conclusions about true causality. Finally, there are procedures available to test the robustness and accuracy of estimated parameters from cross-sectional data (Epskamp et al., 2018c), but unfortunately there are no tools available for time-series data and mlVAR (Aalbers et al., 2019). Hence, we encourage future research to develop methodological procedures to assess the quality of temporal networks.

Despite these constraints, an important quality of the current study is that we have applied a complex methodology and provide all material to replicate the study *via* Open Science Framework. We also share potential methodological issues that future research may encounter and should be aware of in order to move forward in the understanding of this methodology in the paranoia field.

## Conclusion

The current study provided important contributions to the paranoia field by identifying how certain psychological mechanisms such as self-esteem, sadness, feeling close to others, and experiential avoidance are meaningfully related to paranoia. In addition, we provided evidence that psychopathology can be conceptualized as a complex dynamical system and that temporal network analysis is a useful approach to provide novel insight about the complexity of mechanisms implicated in paranoia.

## Data Availability Statement

Publicly available datasets were analyzed in this study. This data can be found here: https://osf.io/7tk4b.

## Ethics Statement

The studies involving human participants were reviewed and approved by the Complutense University of Madrid’s Institutional Review Board and conducted according to the Declaration of Helsinki. The patients/participants provided their written informed consent to participate in this study.

## Author Contributions

AC and CV developed the design. AC and AH carried out the analysis. AC, CV, and AH wrote the first draft of the manuscript. CV, AH, and RB supervised the project. All authors contributed to the article and approved the submitted version.

### Conflict of Interest

The authors declare that the research was conducted in the absence of any commercial or financial relationships that could be construed as a potential conflict of interest.

## Footnotes

^1^https://clinicaltrials.gov/ct2/show/NCT04476771

^2^https://qualtrics.com
