# Supplementary material for: A Temporal Network Approach to Paranoia: A Pilot Study
Source: Front Psychol. 2020 Sep 18;11:544565. doi: 10.3389/fpsyg.2020.544565 (PMC7530190; doi:10.3389/fpsyg.2020.544565)
Supplement: Supplementary file 1 [file Data_Sheet_1.pdf]

Supplementary material

A temporal network approach to paranoia: A pilot study

Alba Contreras[1], Carmen Valiente[1], Alexandre Heeren[2], Richard Bentall[3]

[1] Department of Personality, Assessment and Clinical Psychology, Complutense University, Madrid, Spain

[2]Psychological Sciences Research Institute & Institute of Neuroscience, Université Catholique de Louvain, Louvain-la-Neuve, Belgium

[3]Department of Psychology, University of Sheffield, Sheffield, United Kingdom

Content

1. Table S1. Differences test for demographic, clinical and ESM characteristics between included and excluded participants.

2. Identifying redundant items strategy

3. Table S2. ESM English and Spanish protocol

4. Table S3. Differences test for demographic, clinical and ESM characteristics between participants who use app1 and app2.

5. Table S4. Shapiro-Wilk normality test values.

1. Table S1. Differences test for demographic, clinical and ESM characteristics between included and excluded participants

	Included (n=23)	Excluded (n= 41)	*t/x²*	*p*
Demographic characteristics				
Age in years, mean (SD)	23.78 (6.17)	23.27 (4.29)	0.359	0.69
Sex: Women, n (%)	19 (82.6)	34 (82.9)	0.001	0.97
Single status, n (%)	22 (95.7)	40 (97.5)	0.17	0.67
Education, n (%)			0.43	0.51
Secondary School	3 (13)	8 (19.5)		
Post-secondary	20 (87)	33 (80.5)		
Employed, n (%)			0.06	0.97
Unemployed	15 (65.2)	26 (63.4)		
Part-time employment	5 (21.7)	10 (24.4)		
Full-time employment	3 (13)	5 (12.2)		
Clinical characteristics				
SCL-90-R paranoid ideation, mean (SD)	1.24 (0.94)	1.17 (0.85)	0.31	0.75
SCL-90-R interpersonal susceptibility, mean (SD)	1.57 (0.88)	1.66 (0.80)	-0.41	0.67
SCL-90-R anxiety, mean (SD)	1.10 (0.47)	1.27 (0.70)	-1.11	0.27
SCL-90-R depression, mean (SD)	1.93 (0.76)	1.93 (0.73)	-0.15	0.88
ESM observations				
Number of completed observations, mean (SD)	28.48 (6.58)	8.39 (6.45)	11.86	0.00***
Number of missing beeps, mean (SD)	30.83 (13.90)	48.79 (19.16)	-3.93	0.00***
Number of missing data, mean (SD)	10.70 (12.95)	12.71 (17.33)	-4.85	0.62

Note. SCL-90-R=Symptom Checklist-90-R (Cronbach's α=0.79-0.90); *SD*=Standard Deviation; **p*-value<0.05; ** *p*-value<0.01; *** *p*-value<0.001; Number of missing beeps=the final amount of missing notifications due to technical problems; Number of missing data=notifications that participants did not response.

2. Identifying redundant items

Because some ESM variables were measured with more than one item (see table S2 ESM protocol), we use a data driven approach to identify potential conceptual overlap between them. Following previous research (Bernstein, Heeren, & McNally, 2019), we first tested that the correlation matrix was positive definite, using the *is.positive.definite* function within the *corpcor* R package (Schäfer, Opgen-Rhein, Zuber, Ahdesmäki, Silva,. & Strimmer, 2017). Secondly, we identify nodes which most likely measure the same underlying construct with *goldbricker* function within *networktools* R package (Jones, 2019). The results of the function suggested to reduce self-esteem (2 items) and paranoia (3 items). Therefore, we calculate the average of these items before the analysis.

3. Table S2. ESM English and Spanish protocol

Variable	English Item	Spanish Item
Sadness / Tristeza	1. “At this moment, I feel sad”	1. “En este momento me siento triste”.
Others / Otros	1. “At this moment, I feel close to others”	1. “En este momento, me siento cercano a los demás”.
Experiential Avoidance / Evitación experiencial (EA)	1. “Since the last “beep”, I have tried to avoid negative thoughts and feelings”	1. “Desde el último pitido, he tratado de quitarme de la cabeza pensamientos y sentimientos negativos”.
Paranoia /Paranoia	1. “Since the last “beep”, I have had the impression that I cannot trust people”<br>2. “Since the last “beep”, I have had the impression that people have tried to harm me”<br>3. “Since the last “beep”, I have had the impression that people have criticized me”	1. “Desde el último pitido, he tenido la sensación de que no se puede confiar en la gente”.<br>2. “Desde el último pitido, he tenido la sensación de que la gente ha intentado fastidiarme”.<br>3. “Desde el último pitido, he tenido la sensación de que la gente me ha criticado”.
Self-esteem / Autoestima (SE)	1. “At this moment, I feel useless”<br>2. “At this moment, I feel I can manage issues well”	1. “En este momento, me siento útil”.<br>2. “En este momento, siento que me enfrento bien a los problemas”.

4. Table S3. Differences test for demographic, clinical and ESM characteristics between participants who use app1 and app2.

	app1 (*n*=51)	app2 (*n*=13)	*t/x²*	*p*
Demographic characteristics				
Age in years, mean (SD)	23.27 (4.80)	24.15 (5.90)	-0.56	0.57
Sex: Women, n (%)	43 (84.3)	10 (76.9)	0.39	0.52
Single status, n (%)	50 (98)	12 (92.3)	1.12	0.28
Education, n (%)			0.03	0.84
Secondary School	9 (17.6)	2 (15.4)		
Post-secondary	42 (82.4)	11 (84.6)		
Employed, n (%)			0.62	0.73
Unemployed	32 (62.7)	9 (69.2)		
Part-time employment	13 (25.5)	2 (15.4)		
Full-time employment	6 (11.8)	2 (15.4)		
Clinical characteristics				
SCL-90-R paranoid ideation, mean (SD)	1.26 (0.88)	0.93 (0.76)	1.24	0.21
SCL-90-R interpersonal susceptibility, mean (SD)	1.72 (0.79)	1.25 (0.80)	1.89	0.06
SCL-90-R anxiety, mean (SD)	1.25 (0.64)	1.01 (0.49)	1.26	0.21
SCL-90-R depression, mean (SD)	1.98 (0.76)	1.81 (0.56)	0.76	0.44

Note. SCL-90-R= Symptom Checklist-90-R (Cronbach's α= 0.79-0.90); *SD*= Standard Deviation; **p*-value<0.05; ** *p*-value<0.01; *** *p*-value<0.001

5. Table S4. Shapiro-Wilk normality test values.

Variable	Statistic	*p*
Sad	0.91173	0.000***
SE	0.98427	0.000***
Others	0.96034	0.000***
EA	0.90518	0.000***
Paranoia	0.75771	0.000***

Note: *p-value<0.05; ** *p*-value<0.01; *** *p*-value<0.001

References

Bernstein, E. E., Heeren, A., & McNally, R. J. (2019). Reexamining trait rumination as a system of repetitive negative thoughts: A network analysis. *Journal of Behavior Therapy and Experimental Psychiatry*, *63*(May), 21–27. https://doi.org/10.1016/j.jbtep.2018.12.005

Jones, P. J. (2019). Tools for Identifying Important Nodes in Networks R package. *R Package Version >=3.0.0*.

Schäfer, J., Opgen-Rhein, R., Zuber, V., Ahdesmäki, M., Silva, A. P. D., & Strimmer, K. (2017). Package 'corpcor': Efficient estimation of covariance and (partial) correlation. *R Package Version >=3.0.2*.
